# Comparative Analysis of the Complete Chloroplast Genomes in *Allium* Section *Bromatorrhiza* Species (Amaryllidaceae): Phylogenetic Relationship and Adaptive Evolution

**DOI:** 10.3390/genes13071279

**Published:** 2022-07-19

**Authors:** Junpei Chen, Dengfeng Xie, Xingjin He, Yi Yang, Xufeng Li

**Affiliations:** 1 Key Laboratory of Bio-Resources and Eco-Environment of Ministry of Education, College of Life Sciences, Sichuan University, Chengdu 610065, China; junpeichen123@163.com (J.C.); df_xie2017@163.com (D.X.); yangyi528@scu.edu.cn (Y.Y.); 2 College of Life Sciences, Southwest University, Chongqing 400715, China

**Keywords:** *Allium*, sect. *Bromatorrhiza*, chloroplast genome, comparative analysis, phylogeny, adaptational evolution

## Abstract

With the development of molecular sequencing approaches, many taxonomic and phylogenetic problems of the genus *Allium* L. have been solved; however, the phylogenetic relationships of some subgenera or sections, such as section *Bromatorrhiza*, remain unresolved, which has greatly impeded our full understanding of the species relationships among the major clades of *Allium*. In this study, the complete chloroplast (cp) genomes of nine species in the *Allium* sect. *Bromatorrhiza* were determined using the Illumina paired-end sequencing, the NOVOPlasty de novo assembly strategy, and the PGA annotation method. The results showed that the cp genome exhibited high conservation and revealed a typical circular tetrad structure. Among the sect. *Bromatorrhiza* species, the gene content, SSRs, codon usage, and RNA editing site were similar. The genome structure and IR regions’ fluctuation were investigated while genes, CDSs, and non-coding regions were extracted for phylogeny reconstruction. Evolutionary rates (Ka/Ks values) were calculated, and positive selection analysis was further performed using the branch-site model. Five hypervariable regions were identified as candidate molecular markers for species authentication. A clear relationship among the sect. *Bromatorrhiza* species were detected based on concatenated genes and CDSs, respectively, which suggested that sect. *Bromatorrhiza* is monophyly. In addition, there were three genes with higher Ka/Ks values (*rps2*, *ycf1*, and *ycf2*), and four genes (*rpoC2*, *atpF*, *atpI*, and *rpl14*) were further revealed to own positive selected sites. These results provide new insights into the plastome component, phylogeny, and evolution of *Allium* species.

## 1. Introduction

*Allium* L. is one of the largest monocotyledonous genera, with more than 1000 species worldwide currently [1]. This genus is of great economic value, including many important vegetables, for example, garlic, leek, onion, and shallot [2,3], and many of them are also cultivated as spices, medicinal, or ornamentals plants, such as *Allium wallichii* Kunth [4,5] and *Allium stipitatum* Regel. [6]. A large number of previous phylogenetic studies have considerably improved our comprehension of the taxonomic relationships and evolutionary processes of the genus [7,8,9,10,11,12,13], and three distinct evolutionary lineages with 15 subgenera were recognized [7,8,10]. Despite many studies on this genus, the taxonomical and phylogenetic relationships of some subgenera or sections still need to be resolved, for example, *Allium* section *Bromatorrhiza* Ekberg.

The sect. *Bromatorrhiza* belongs to the subgenus *Amerallium* Traub of the genus *Allium* and is located in the first evolutionary lineage according to previous studies, containing about eight species and two varieties [3,10,14]. According to Flora of China, species in this section mainly grow on wet grassy slopes, near rocks or on the edge of forests, widely distributed in the Himalayas-Hengduan Mountains region and Qinghai-Tibetan Plateau [3,10], and are characterized by thickened fleshy roots, which can distinguish them from most other *Allium* species. Six species of this section are endemic to China, and all occur in the Hengduan Mountains, with narrow distribution areas. Another four species named *A. wallichii*, *Allium macranthum* Baker, *Allium fasciculatum* Rendle, and *Allium hookeri* Thwaites extend their distribution ranges from southern Tibet, Sichuan, and Yunnan province (China) to the neighboring countries of Nepal, Bhutan, and India [3].

In traditional taxonomy, the sect. *Bromatorrhiza* is a controversial group. It was originally described by *Ekberg* L., who considered sect. *Bromatorrhiza* belongs to the subgenus *Bromatorrhiza* Ekberg, which was mainly based on the occurrence of fleshy roots as storage organs and the absence of true storage bulbs or rhizomes [15]. However, because the viewpoint of the subgenus *Bromatorrhiza* was artificial and not widely accepted [16,17], the taxonomic and phylogenetic position of sect. *Bromatorrhiza* remains uncertain. Xu reclassified this section on the basis of morphological features and suggested that more than 10 species belong to this section [14], also including *Allium trifurcatum* (F. T. Wang & T. Tang) J. M. Xu and *Allium cyathophorum* var. *farreri* Stearn. However, subsequent phylogenetic studies detected that these two species and *Allium cyathophorum* Bur. et Franch. are not recommended for members of sect. *Bromatorrhiza* [7,10,11,17,18,19], which was further confirmed by the basic chromosome number, with x = 7, 10, or 11 in sect. *Bromatorrhiza* species while in *A. trifurcatum*, *A. cyathophorum*, and *A. cyathophorum* var. *farreri*, x = 8 [20,21,22,23,24]. Meanwhile, *A. cyathophorum* var. *farreri* was revised as *Allium farreri* Stearn [25]. In addition, Huang [26] first proposed the idea of the *Allium hookeri* complex, which includes *Allium hookeri* Thwaites, *Allium chienchuanense* J. M. Xu, *Allium omeiense* Z. Y. Zhu, *Allium xiangchengense* J. M. Xu, *Allium guanxianense* J. M. Xu, and *Allium hookeri* Thwaites var. *muliens* Airy-Shaw, based on the same basic chromosome number x = 11 and similar morphological features [27]. However, there is still no other evidence to support this viewpoint, especially the lack of convincing molecular evidence. Although these previous studies have greatly promoted the taxonomy and phylogeny of this section, the species relationship within this section species and their relatives is still unclear and needs to be further investigated.

In angiosperm species, chloroplasts are important and common organelles for photosynthesis [28]. This is due to their unique tetrad structure, high conservation in terms of gene order and gene content [29], and lower substitution rates than nuclear DNA (especially in regions of inverted repeats) [29,30,31], providing a promising solution to phylogenetic uncertainty, especially for taxonomically complex groups [32,33]. Furthermore, the complete chloroplast (cp) genome can be used for screening of DNA barcode sequences [34,35,36], species divergence time estimation [37], evolutionary rate calculation, and environment adaptive analysis [38,39].

In recent years, many complete chloroplast genomes have been used in *Allium* studies [37,39,40,41,42,43,44]. In this study, the plastomes of all sect. *Bromatorrhiza* species were collected except for *A. guanxianense*. Meanwhile, the chosen three related species (*A. trifurcatum*, *A. cyathophorum*, and *A. farreri*), a homogeneous distribution species (*Allium kingdonii* Stearn) [3], and two other species (*Allium ursinum* L. and *Allium monanthum* Maxim.), also located in the first evolutionary lineage [7,10], were used to perform comparative chloroplast genomes analysis. Finally, the cp genomes of 9 sect. *Bromatorrhiza* species were combined with another 34 *Allium* species and 2 outgroups, aiming to (1) clarify the cp genomes’ structural characteristics of sect. *Bromatorrhiza* species and their relatives, (2) reconstruct and analyze the phylogenetic relationships of sect. *Bromatorrhiza*, and (3) investigate the adaptive evolution of species in this section.

## 2. Materials and Methods

### 2.1. Plant Sample Collection, DNA Extraction, and Complete Genome Sequencing

All the species of sect. *Bromatorrhiza* proposed by Li et al. [10] were sampled except for *A. guanxianense*, which we were unable to collect because of geological hazards that have destroyed its habitat. Other probably related taxa to sect. *Bromatorrhiza* species were also selected to reassess their relationships. Fresh and healthy leaves of single individuals of nine sect. *Bromatorrhiza* species were collected from each field site (Table S1). All voucher specimens in this paper were stored at the Sichuan University Herbarium (SZ). Total genomic DNA was extracted from leaves, which were preserved by drying on silica gel, according to the operating instructions of the Plant Genomic DNA Kit (Tiangen Biotech, Beijing, China). Sequencing was then performed at Novogene (Beijing, China) using the Illumina Novaseq 6000 platform (Illumina, San Diego, CA, USA) and with the Novaseq 150 sequencing strategy.

### 2.2. Chloroplast Genome Sequence Assembly and Annotation

The data obtained were first removed from the connectors and low-quality reads and then assembled via the organelle assembler NOVOPlasty 2.7.1 [45] with the parameters of the genome range (145,000–165,500) and k-mer 39. The cp genome of *Allium cepa* L. was used as the reference (GenBank accession No. KM088014), and *rbcL* chloroplast gene sequences from different species were used as seeds to assemble the plastomes. The *rbcL* gene of *A. hookeri* was used as the seed for *A. hookeri*, *A. hookeri* var. *muliens*, *A. xiangchengense*, *A. chienchuanense*, and *A. omeiense*. For *A. wallichii*, *A. wallichii* var. *platyphyllum*, *A. macranthum*, and *A. fasciculatum*, their seeds were provided by their *rbcL* sequences (GenBank accession in Table 1). Other parameters were left as default values (see NOVOPlasty README.md). *A. cepa* (KM088014) was used as a reference for comparison with the assembled genome sequences using Geneious R11 [46] (http://www.geneious.com; accessed on 21 October 2021) to choose the best option, which was then annotated by PGA [47]. Based on comparison with other homologous genes in the reference chloroplast genomes, conflicting annotations were then manually corrected in Geneious R11. Meanwhile, all identified tRNAs were further verified by tRNAScan-SE v2.0.6 [48]. Finally, the physical maps of the genome were drawn using OGDRAW v 1.3.1 [49], and the GC content of the whole cp genome, IR, LSC, and SSC regions were compared among the species by Geneious R11. The nine annotated plastid genomes were submitted to GenBank, and their accession numbers are listed in Table 1.

The chloroplast genome sequences of the other six species (*A. ursinum*, *A. kingdonii*, *A. monanthum*, *A. trifurcatum*, *A. farrer*, and *A. cyathophorum*) were downloaded from NCBI (accession numbers listed in Table S1), five of which were submitted by our team, except for *A*. *ursinum*, and then re-annotated under the same conditions for subsequent comparative analyses in this study.

### 2.3. Contraction and Expansion of IRs and Repeat Content

The program IRscope [50] (https://irscope.shinyapps.io/irapp/, accessed on 30 October 2021) was used to compare and visualize the boundaries between the LSC, IR, and SSC regions of the 15 species. The simple sequence repeats (SSRs) were identified using Perl script MISA [51] (http://pgrc.ipk-gatersleben.de/misa/, accessed on 22 October 2021), including mono-, di-, tri-, tetra-, penta-, and hexanucleotides with the following repeat threshold settings of 10, 5, 4, 3, 3, and 3, respectively. The online REPuter software was used to identify the tandem repeat sequences [52] (https://bibiserv.cebitec.uni-bielefeld.de/reputer/manual.html, accessed on 3 November 2021) with the following parameters: (1) a repeat size of over 30 bp; (2) sequence identity between 2 repeats of over 90%; and (3) Hamming distance = 3.

### 2.4. Codon Usage Bias Analysis and RNA Editing Sites

The protein-coding genes were extracted from the 15 plastomes for codon analysis. Codon usage bias analysis and calculation of the RSCU [53] values were performed in the program CodonW v1.4.2 (https://sourceforge.net/projects/codonw/, accessed on 28 November 2021). A total of 52 shared protein-coding genes (CDS) were screened out from 15 species following the guidance of removing CDS smaller than 300 bp [54] and the overlapping genes. Five important indices were used to assess the extent of the codon usage bias, including the codon bias index (CBI), frequency of optimal codons (Fop), codon adaptation index (CAI), GC content of the synonymous third codons positions (GC3s), and the effective number of codons (Nc). The base compositions for protein-coding genes were estimated by MEGA6 [55]. Potential RNA editing sites were identified based on the strategy of a cut-off value of 0.8 in the online program PREP Suite [56] ( http://prep.unl.edu/, accessed on 9 April 2022).

### 2.5. Sequence Divergence and Nucleotide Diversity Analysis

Using *A. wallichii* as a reference, the comparison results of the 15 cp genomes were visualized with the Shuffle-LAGAN mode of mVISTA [57]. Meanwhile, the default settings of Mauve v2.4.0 [58] were used to identify large structural changes such as gene order rearrangements, inversions, and insertions in the chloroplast genomes. All plastid genome sequences were aligned in Geneious R11 with MAFFT v7 [59]. Subsequently, indels and SNPs were counted and positioned using the “Find Variations/SNPs”. The genetic distance of all sequences was calculated using MEGA6 [55]. For the 15 aligned cp genome sequences, the Pi values of the compared chloroplast genomes were calculated using DnaSP v5 [60] in the sliding window for DNA polymorphism analysis. The parameters were set as follows: (1) windows size of 600 bp; (2) step size of 200 bp.

### 2.6. Phylogenetic Tree Analysis

To infer phylogenetic relationships within sect. *Bromatorrhiza* and its relatives, the 15 cp genomes analyzed above were compared to the other 28 *Allium* species (download from NCBI, Table S2), choosing *Narcissus poeticus* Sm. and *Agapanthus coddii* F. M. Leight as outgroups based on previous phylogenetic studies [37,39,44]. All chloroplast genome sequences were aligned as a whole with MAFFT v7 [59]. The following three datasets were used for phylogenetic analysis: (A) All genes shared between species, excluding duplicates and largely divergent sequences; (B) all the single-copy CDS sequences / protein-coding genes; (C) all the non-coding regions. The best-fit models for both datasets were selected by MrModeltest v3.7 [61]. Maximum likelihood (ML) analyses were performed using RAxML 8.2.8 [62] with the GTR + G model and 1000 bootstrap replicates. Bayesian inference (BI) was performed in Mrbayes v3.2 [63] under the GTR+I+G model with Markov chain Monte Carlo (MCMC) analysis running for 1 × 10^8^ generations and one tree sampled every 1000 generations, respectively. The first 25% were discarded as burn-in and the remaining trees were used to establish a 50% majority-rule consensus tree. When the average standard deviation of the splitting frequency remained below 0.001, it was considered that stationarity was achieved.

### 2.7. Positive Selection Analysis

In total, 80 protein-coding sequences (CDS) larger than 300 bp in each chloroplast genome of sect. *Bromatorrhiza* were used to analyze the non-synonymous (Ka) and synonymous (Ks) nucleotide substitution rates and their ratios (ω = Ks/Ks) to measure the selective pressure. First of all, MAFFT v7 [59] was used to compare these 80 CDS and manually adjusted by MEGA6 [55]. Then, pairwise Ka/Ks ratios of the nine plastomes were calculated in KaKs_Calculator 2.0 [64]. Genes with ω < 1, ω = 1, and 1  <  ω were considered under purifying selection (negative selection), neutral selection, and positive selection, respectively [65]. The ω ≥ 20 or NA indicates that the gene has few nonsynonymous sites/substitutions and was not considered in our analysis. The log-likelihood values were calculated and tested according to Lan et al. [66]. The Bayesian Empirical Bayes (BEB) [67] method was applied to compute the posterior probabilities of amino acid sites to identify whether these specific sites were under positive selection (codon sites with a high posterior probability) [68]. A gene with a test *p*-value < 0.05 and with positively selected sites was considered a positively selected gene (PSG).

## 3. Results

### 3.1. Genome Features of sect. Bromatorrhiza

**Figure 1:**
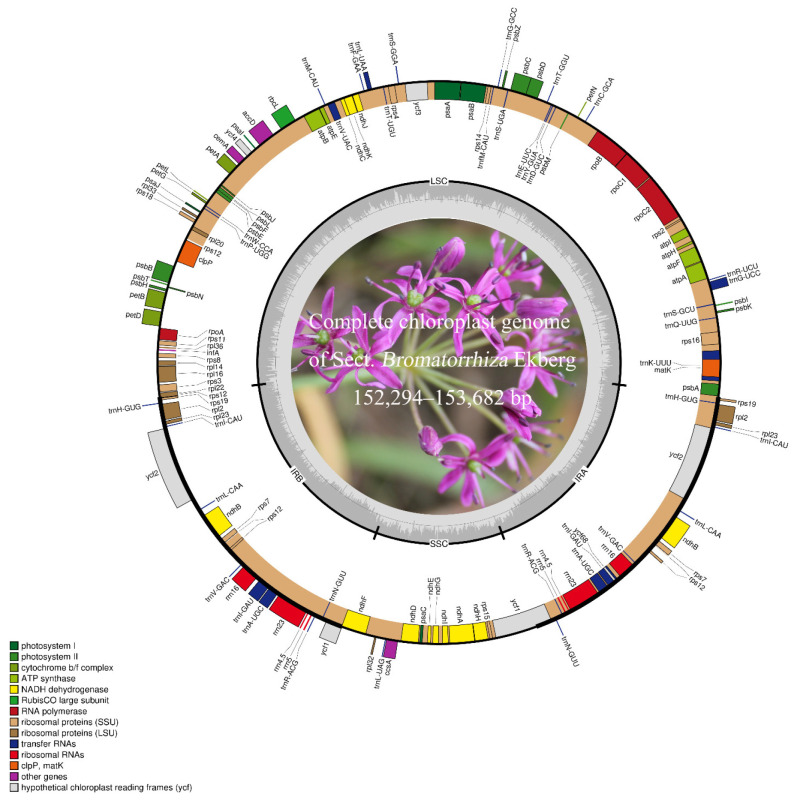
Maps of nine sect. *Bromatorrhiza* plastomes. The outer circle shows genes transcribed counterclockwise while the inner ones are those transcribed clockwise. Colored bars indicate the different functional regions. The dark grey area in the inner circle indicates the GC content while the light grey area represents the AT content. LSC: large single-copy region; SSC: small single-copy region; IR: inverted repeat region.

The complete chloroplast genomes of all sect. *Bromatorrhiza* species are typical quadripartite structures and include four sections as a large single-copy (LSC) region, a pair of IR regions (IRa and IRb), and a small single-copy (SSC) region (Figure 1). The total cp genome size ranged from 152,294 (*A. xiangchengense*) to 153,682 bp (*A. hookeri*) (Table 1), the IR ranged from 26,256 (*A. xiangchengense*) to 26,825 bp (*A. chienchuanense*), and the LSC and SSC length ranged from 82,168 (*A. macranthum*) to 82,700 bp (*A. hookeri*) and 17,121 (*A. wallichii* var. *platyphyllum*) to 17,606 bp (*A. macranthum*), respectively. The GC content analysis showed that the overall GC contents ranged from 37.0 to 37.1% in the plastomes of sect. *Bromatorrhiza* species, which were significantly higher in the IR regions (42.5~42.7%) than in the LSC regions (34.8~34.9%) and the SSC regions (30.0~30.4%). Complete chloroplast genomes of sect. *Bromatorrhiza* have been submitted to GenBank (Table 1).

Each of the cp genomes encodes 134 unique genes of the same gene order and gene clusters, including 88 protein-coding genes (PCGs), 38 tRNA, and 8 rRNA genes (Table 1). The SSC region contains 11 PCGs (e.g., *ndhF*, *rpl32*, and *ccsA*) and 1 tRNA (*trnL-UAG*) while the LSC region contains 62 PCGs and 21 tRNAs. Fifteen genes (*trnK-UUU*, *rps16*, *trnG-UCC*, *atpF*, *rpoC1*, *trnL-UAA*, *trnV-UAC*, *petB*, *petD*, *rpl16*, *rpl2*, *ndhB*, *trnI-GAU*, *trnA-UGC*, and *ndhA*) contain one intron while three genes (*rps12*, *ycf3*, and *clpP*) contained two introns (Figure 1). Notably, the *rps12* gene is a trans-spliced gene. For the detailed genome components and structures, please see Figure 1 and Table S3.

### 3.2. Comparative Analysis of the Chloroplast Genome Structure of sect. Bromatorrhiza

**Figure 2:**
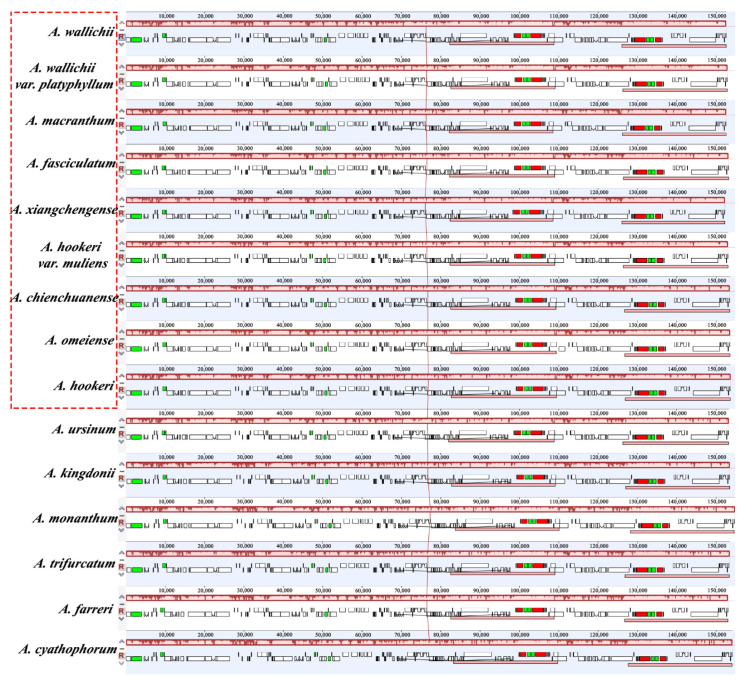
MAUVE alignment of chloroplast genomes of nine sect. *Bromatorrhiza* species and their relatives using Geneious R11. Local collinear blocks are represented by blocks of the same color and linked within each of the alignments. Box in red is for sect. *Bromatorrhiza* species.

**Figure 3:**
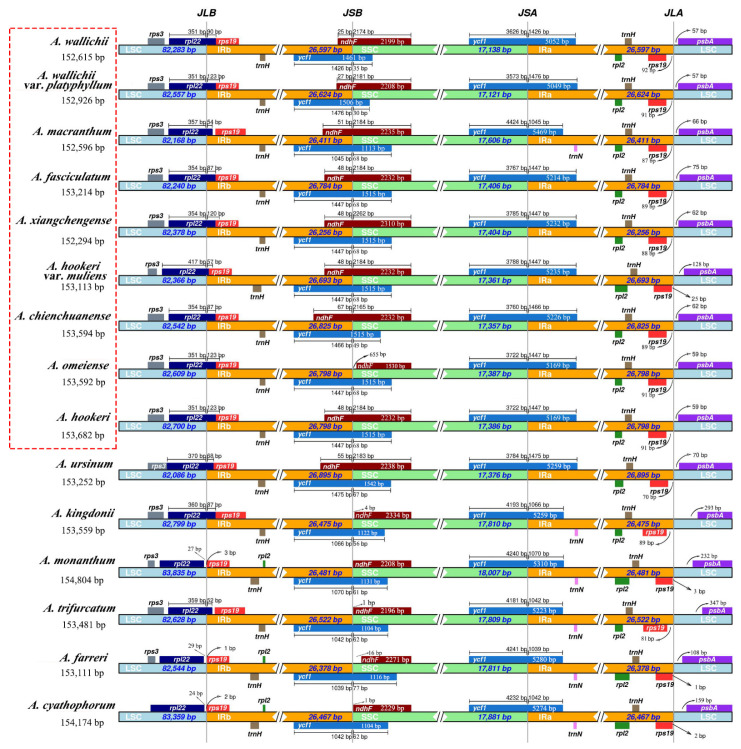
Comparison of the LSC, SSC, and IR junction of plastid genomes between the nine sect. *Bromatorrhiza* species and their relatives. JLB indicates the junction line between LSC and IRb; JSB indicates the junction line between SSC and IRb; JSA indicates the junction line between SSC and IRa; JLA indicates the junction line between LSC and IRa. Box in red is for sect. *Bromatorrhiza* species.

The Mauve alignment revealed that there was no rearrangement in the plastid genomes of sect. *Bromatorrhiza* species and their relatives (Figure 2). The IR boundaries of the 15 chloroplast genomes were compared (expanded or contracted) using a comparative analysis of the genes around the boundaries (Figure 3). The results revealed that some genes straddled or were close to the boundary of the IR regions, such as *rps3*, *rpl22*, and *rps19* (Figure 3). The *rpl22* genes of *A. monanthum*, *A. cyathophorum* var. *farreri*, and *A. cyathophorum* were found to be located in the LSC region only, whereas those of the other 12 species spanned the LSC/IRb boundary (JLB line), with 54–123 bp situated in the IR region (Figure 3). This may be due to the expansion occurring in the IR region and the contraction in the LSC region. Furthermore, the *ycf1* gene straddles the SSC/IRa boundary (JSA line), most of which are located in the SSC region, with 1045–1476 bp in the IR region. Interestingly, among the sect. *Bromatorrhiza* plastid genomes, only the *ndhF* of *A. omeiense* is located entirely in the SSC region and adjacent to the SSC/IRb boundary (JSB line) by 655 bp, which in other species spans the SSC/IRb boundary (JSB line), with 25–67 bp located in the IR regions (Figure 3).

**Figure 4:**
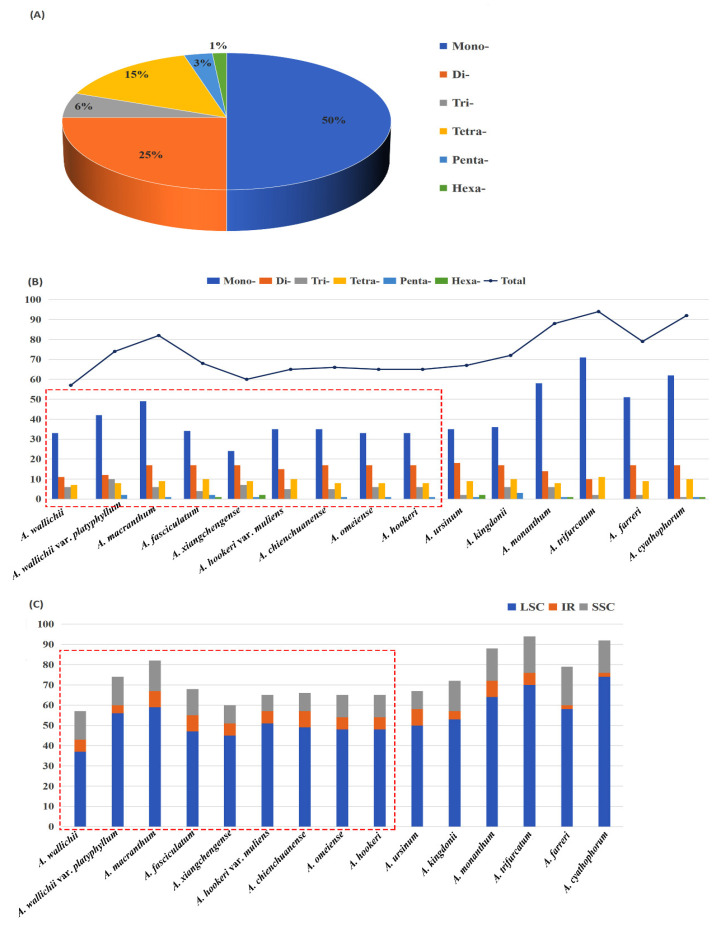
Analyses of the simple sequence repeats (SSRs) in 15 plastomes: (**A**) proportion of different repeat types in the plastid, (**B**) numbers of different repeat types, (**C**) presence of SSRs in LSC, SSC, and IR. Boxes in red are for sect. *Bromatorrhiza* species.

**Figure 5:**
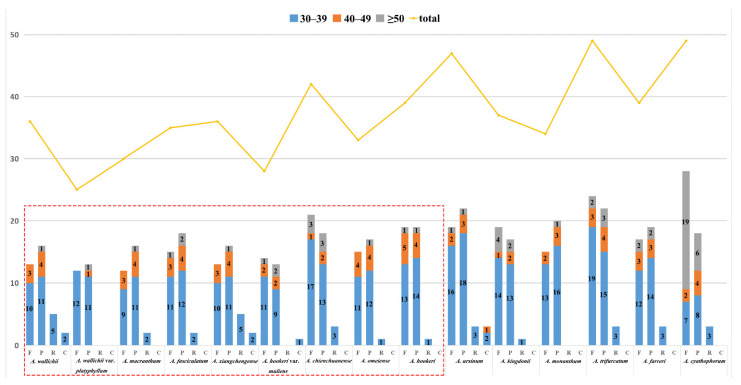
Variation in the distribution of forward (F), reverse (R), complementary (C), and palindromic (P) repeats and the number of different repeats in the chloroplast genome of 15 plastomes. Box in red is for sect. *Bromatorrhiza* species.

The results showed the total number of SSR for the sect. *Bromatorrhiza* plastomes ranged from 57 (*A. wallichii*) to 82 (*A. macranthum*), and the distribution modes of SSRs were similar among the 15 plastid genomes (Table S4: Figure 4). Mononucleotides were the most frequent in the SSRs (40.00–59.76%), followed by dinucleotides (16.22–28.33%) and trinucleotides (5.88–13.51%) (Figure 4) while some species lacked penta- and hexanucleotides, such as *A. wallichii* and *A. hookeri* var. *muliens* (Table S4: Figure 4B). Furthermore, A/T bases were the major components for all identified SSRs in the 15 plastomes. In addition to SSRs, 608 long repeats (forward, palindromic, reverse, and complement) were identified using REPuter (Table S5: Figure 5). Among sect. *Bromatorrhiza* species, the most common types of repeats were forward (F) and palindromic (P) repeats, and complement (C) repeats were only identified in *A. wallichii*, *A. hookeri* var. *muliens*, and *A. xiangchengense*. Among sect. *Bromatorrhiza* species, *A. chienchuanense* showed the maximum repeat number (42), and *A. wallichii* var. *platyphyllum* possessed the minimum repeat number (25). Given the lengths of repeats, most repeats ranged from 30 to 39 bp (Table S5: Figure 5).

### 3.3. Codon Usage Bias and RNA Editing Site Analysis

**Figure 6:**
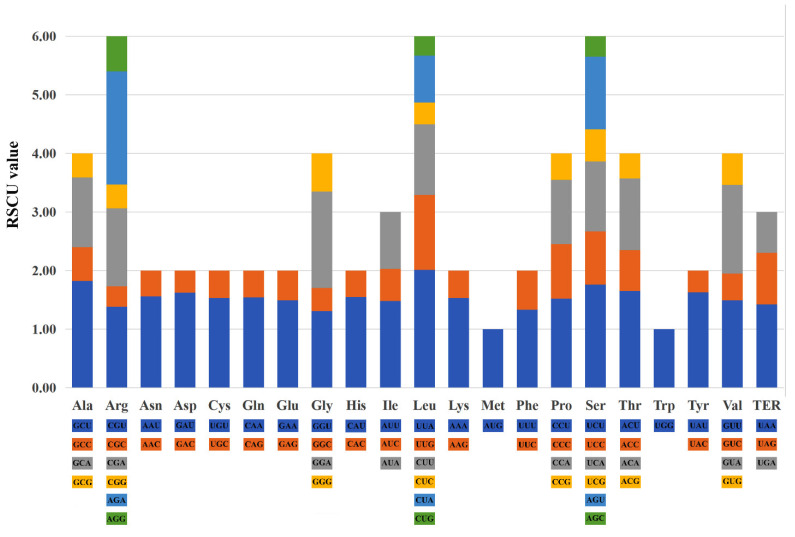
Codon content of 20 amino acids and stop codons in the 9 sect. *Bromatorrhiza* species.

**Figure 7:**
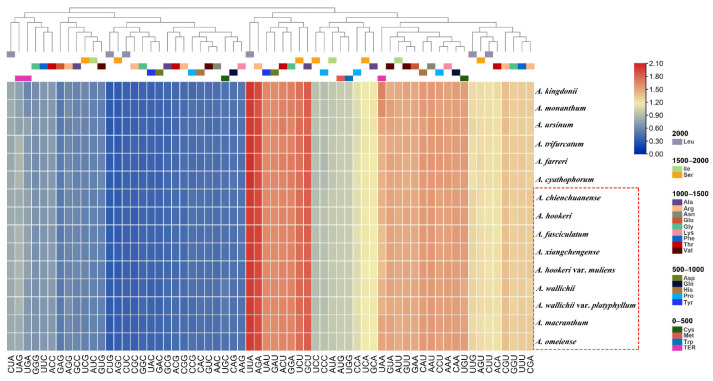
The RSCU values of all merged protein-coding genes for 15 plastomes. In the colored boxes, higher values in red indicate higher RSCU values and, conversely, higher values in blue indicate lower RSCU values. Box in red is for sect. *Bromatorrhiza* species.

The total sequence sizes of the 52 protein-coding genes for the codon analysis were 62,814–63,678 bp in the 9 sect. *Bromatorrhiza* plastomes. These protein sequences encoded 20,938–21,320 codons (Table S6). The majority of the amino acids exhibited codon preferences, with the exception of Met and Trp (Figure 6). Leu was encoded by the highest number of codons (2154–2202) while Cys was the lowest (241–246). The RSCU values of all codons are shown in Figure 7 (Table S6). Values in red and blue represent higher and lower RSCU values, respectively. The RSCU values showed that roughly half of the codons were used more frequently. There were 30 codons used frequently with RSCU > 1, all ending in A/T except for UUG, and two codons (ATG and TGG) had no usage bias (RSCU = 1) (Table S6). The CAI, CBI, FOP, Nc, GC3s, and GC% values are summarized in Table S7.

Additionally, potential RNA editing sites for all the protein-coding genes were performed by the PREP suite in the sect. *Bromatorrhiza* plastomes. In total, 548 RNA editing site were recognized. The number of editing sites ranged from 59 (*A. omeiense*) to 63 (*A. wallichii* var. *platyphyllum*) (Table S8). The *ndhB* gene had the highest number of RNA editing sites (13–14) (Figure S1B). All recognized RNA editing sites were cytosine to uracil (C-U) transitions, most of which were located at the second codon position (48–52), followed by the first codon position (11–12), with no transitions at the third codon position (Figure S1A). The amino acid conversion of serine to leucine (S-L) occurred most frequently (265).

### 3.4. Sequence Divergence Analysis

**Figure 8:**
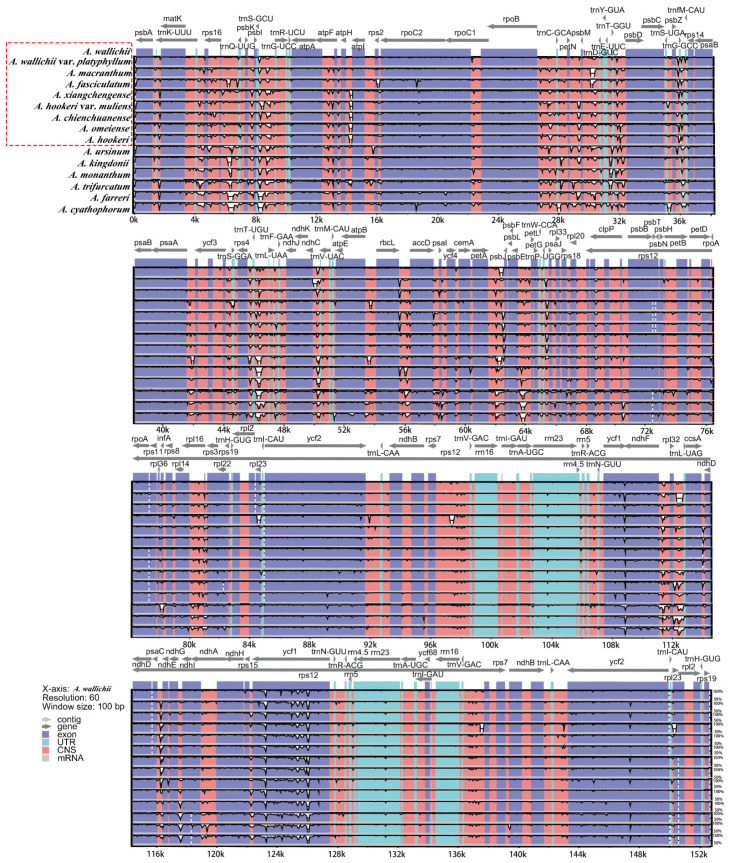
VISTA-based sequence identity plot of 15 chloroplast genomes with *A. wallichii* as a reference. The percentage identity ranging from 50 to 100% is represented by the vertical scale. Coding and non-coding regions are colored in purple and pink, respectively. Box in red is for sect. *Bromatorrhiza* species.

**Figure 9:**
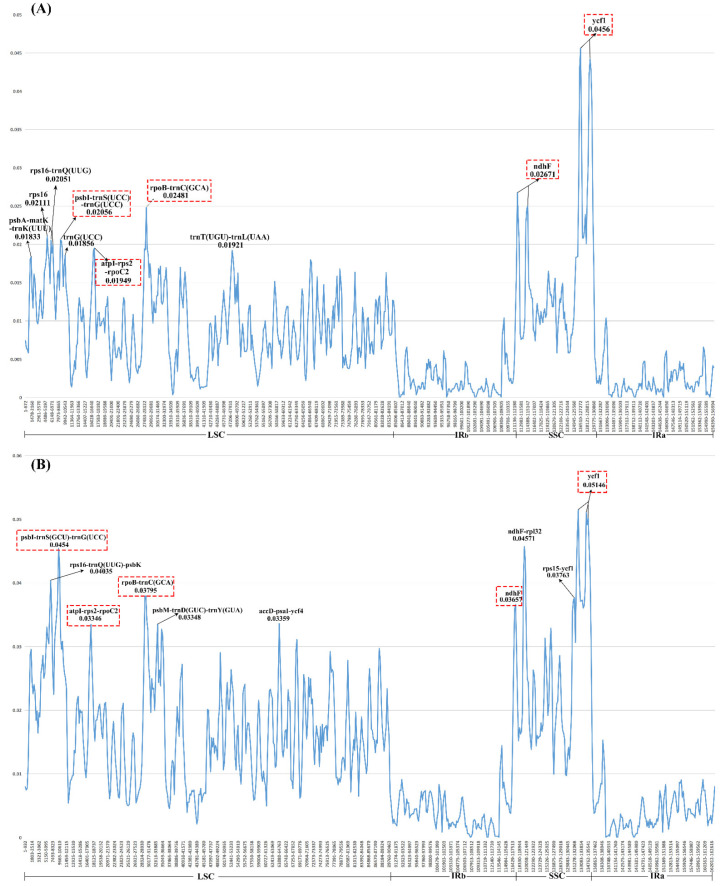
The nucleotide diversity of the plastid genome of (**A**) the 9 sect. *Bromatorrhiza* species and (**B**) 15 allied species in *Allium*. Ten regions with the highest Pi values were labeled. LSC indicates the large single-copy region; IR indicates the inverted repeat region; SSC indicates the small single-copy region.

Sequence divergence of the sect. *Bromatorrhiza* and its relatives was analyzed using mVISTA and DnaSP, and hypervariable regions further detected and sequence identity plots constructed (Figure 8), with the annotated chloroplast genome of *A. wallichii* as the reference. The results showed that the number of genes and sequences in the IR regions was relatively conserved and less divergent than in the LSC and SSC regions (Figure 9). A number of these highly variable regions were found in non-coding sequences (CNS) (Figure 8). We also calculated the genetic distance of the 15 plastomes (Table S9). The pairwise genetic distance ranged from 0.000241 (*A. omeiense* and *A. hookeri*) to 0.023899 (*A. ursinum* and *A. cyathophorum*), and the pairwise genetic distance among sect. *Bromatorrhiza* species ranged from 0.000241 (*A. omeiense* and *A. hookeri*) to 0.011184 (*A. wallichii* and *A. hookeri* var. *muliens*). A total number of 1670 SNPs (1176) and indels (494) were detected among the 15 plastomes, most of which were from non-coding regions (1168) (Table S10).

Moreover, the nucleotide diversity (Pi) of the chloroplast genomes of sect. *Bromatorrhiza* and their relatives were calculated to evaluate their sequence divergence levels. For all 15 plastomes in this study, Pi values in the LSC region ranged from 0.00213 to 0.0454, with a mean of 0.0172, and from 0.02639 to 0.05146 in the SSC region, with an average of 0.0263, while in the IR regions, Pi values changed from 0.00975 to 0.01605 with an average value of 0.00412 (Figure 9B). The low Pi values in the IR region indicated that there were fewer mutations in the IR region and that it was highly conserved. The Pi values of 9 and 15 plastomes were compared, respectively, and 10 regions with high Pi values were obtained in each group, 5 of which were identical in the 2 sets of results (e.g., *ycf1*, *pbI-trnS-trnG*, and *rpoB-trnC*) (Figure 9).

### 3.5. Phylogenetic Analysis

**Figure 10:**
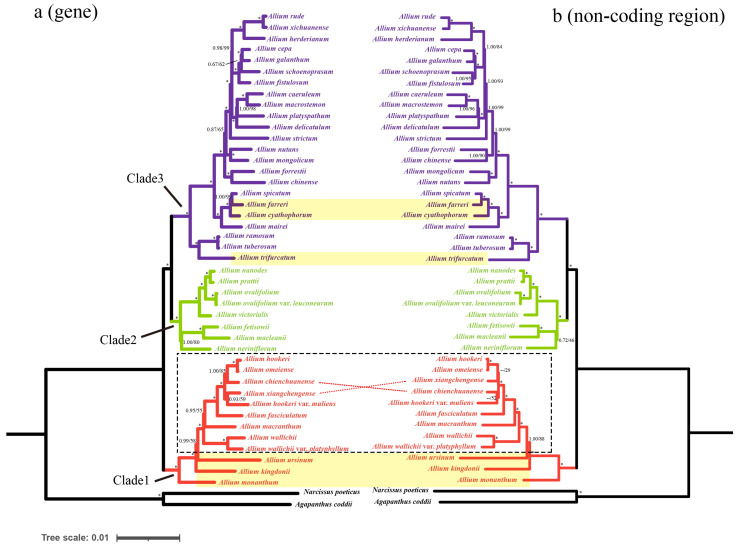
Phylogeny of the 45 taxa inferred from maximum likelihood (ML) and Bayesian inference (BI) analyses based on shared genes and non-coding regions. The numbers to the left of the slashes on the branches show the posterior probabilities (PPs) according to Bayesian inference, and those to the right show the bootstrap values (BS) obtained by maximum likelihood analyses. * Maximum support of 1.00/100—no statistical support. Three evolutionary lineages (Clade 1–3) are marked with different colors: Clade 1, red; Clade 2, green; Clade 3, purple. The nine sect. *Bromatorrhiza* species are located in the black dotted box, and the other six species compared above are shaded in yellow.

Most of the genes, CDSs, and non-coding regions were selected as three datasets to investigate the phylogenetic relationships. The phylogenic trees derived from Bayesian inference were topologically similar to those from the ML analyses, with high bootstrap support values (BS  >  90%) and strong posterior probabilities (PP  =  1) (Figure 10). All *Allium* species clustered into three lineages (clade 1–3) and the nine sect. *Bromatorrhiza* species clustered into a monophyletic clade with strong support (Figure 10). *A. wallichii* and *A. wallichii* var. *platyphyllum* formed a sister relationship with the other seven species and obtained strong support values (PP = 1, BS = 100%, Figure 10 and Figure S2). *A. fasciculatum* formed a good sister relationship with the *A. hookeri* complex (containing *A. hookeri*, *A. chienchuanense*, *A. omeiense*, *A. xiangchengense*, and *A. hookeri* var. *muliens*) (PP = 1, BS = 100%, Figure 10 and Figure S2). While in the *A. hookeri* complex, *A. hookeri* was a sister to *A. omeiense*, and then, *A. chienchuanense* or *A. xiangchengense* exhibited an unstable relationship with *A. hookeri* and *A. omeiense*. Additionally, the related species of *A. cyathophorum*, *A. farreri*, and *A. trifurcatum* are located in the third lineage of *Allium*, and only *A*. *kingdonii* belongs to the first lineage of the *Allium* (Figure 10 and Figure S2).

### 3.6. Adaptive Evolution Analysis

**Figure 11:**
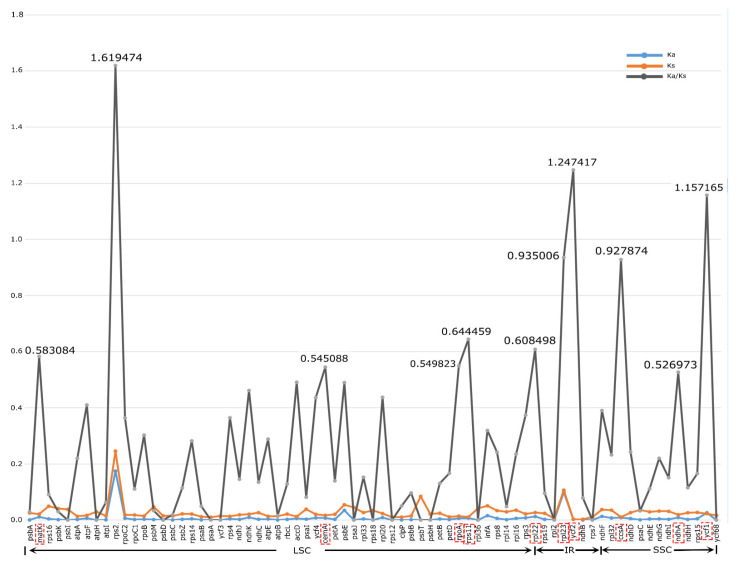
Selective pressure of 80 protein-coding genes in the 9 sect. *Bromatorrhiza* species. Ka: rate of non-synonymous substitution; Ks: rate of synonymous substitution. Ka/Ks values > 0.5 with red boxes.

In total, 80 shared CDSs were filtered and used for Ka (synonymous substitution rates) and Ks (nonsynonymous substitution rates) calculation and positive selection analysis among sect. *Bromatorrhiza* species. The results showed that *rps2*, *rpl23*, *psbE*, and *ycf1* had relatively high average Ka values (Ka > 0.02) while *rps2*, *rpl23*, *psbT*, *psbE*, and *infA* had comparatively high average Ks values (Ks > 0.05) (Figure 11: Table S11). It was observed that *rps2* had the highest average Ka/Ks ratio of 1.619474, followed by *ycf2* (1.247417) and *ycf1* (1.157165), and most CDSs had low Ka/Ks values (less than 0.65). The Ka/Ks analysis at the species level of sect. *Bromatorrhiza* and the sequences used were obtained by concatenating all CDSs. The results showed that the Ka/Ks ratios ranged from 0.25289 (*A. omeiense* vs. *A. hookeri*) to 0.47372 (*A. fasciculatum* vs. *A. hookeri*), with an average ratio of 0.37514 (Figure S3). Finally, positive selection analysis was also performed for the first lineage of *Allium* [10], the lineage in which the sect. *Bromatorrhiza* species positioned. All *p*-values were not significant in these CDSs; however, four CDSs were found with positively selected sites in the BEB test (Table S12). Among them, three CDSs only had one positive selective site (*atpF*, *atpI*, and *rpl14*) while *rpoC2* possessed three positive selective sites (Table S12).

## 4. Discussion

### 4.1. Complete Chloroplast Genome Structure

In this study, we described and compared the genome structure of the sect. *Bromatorrhiza* species with their relatives. The results showed that the cp genome of sect. *Bromatorrhiza* species displayed a typical quadripartite structure. The gene order and structures were similar to those of higher plants, implying a highly conserved structure of the chloroplast genome (Table 1, Figure 1). Similar results have been reported previously for many cp genomes [69,70]. Previous studies have shown that the chloroplast genome size of *Allium* (Amaryllidaceae, Allioideae) species ranged from 145,819 to 157,735 bp [37,39,42,43,69,71], and species of sect. *Bromatorrhiza* were scattered within this range. According to previous research, changes in the size of the chloroplast genomes may be caused by gene deletion [72], variation in the intergenic region [73], and expansion or contraction of the IR regions [74,75]. Comparative analysis of IR boundaries showed that the distribution of genes at the SC/IR boundaries in the different plastid genomes of sect. *Bromatorrhiza* were similar, with only some differences in individual genes, e.g., the *ndhF* gene straddled the JSB line in other species of sect. *Bromatorrhiza*, whereas in *A. omeiense*, it was located exclusively in the SSC region, and the same situation was also found in previous studies (*A. kingdonii*, *A. monanthum*, *A. trifurcatum*, *A. farreri*, and *A. cyathophorum*) [37,39,41,44,69]. In this study, the change in the chloroplast genome size in sect. *Bromatorrhiza* might be associated with changes in the IR/SC boundaries (Figure 3).

### 4.2. cp Genome Sequence Variation and Potential DNA Barcode Markers

Some regions with repetitive sequences lead to slip-strand mismatches and intramolecular recombination and are regarded as being accountable for most indel mutations [76]. Microsatellites (SSRs) have been widely used as molecular markers to analyze genetic diversity, population structure, and biogeography due to their polymorphic characteristics [77,78,79]. Among the nine sect. *Bromatorrhiza* plastomes, the most abundant SSRs were mononucleotides, and the SSR number in LSC was more than that in the SSC and IR regions (Figure 4). This phenomenon has also been reported in Liliaceae [80] and Apiaceae [70,81]. The most likely explanation for the highest number of SSRs in LSC was the fact that LSC was longer than SSC and IRs. Meanwhile, according to previous studies, these SSRs can be applied as molecular markers for the study of genetic diversity in sect. *Bromatorrhiza* species [82,83].

A comparison of the nine cp genomes showed a high degree of synteny (Figure 2). The results of both the mVISTA and sliding window analysis showed that the two IR regions were more conserved than the SC regions (Figure 8) and that the non-coding regions showed greater variation than the coding regions, possibly due to copy number differences in the inverted repeat sequences caused by gene conversion [84,85].

A DNA barcode has been defined as a short DNA sequence with sufficient variation to distinguish a species within a specific taxonomic group [86], phylogenetic relationships, and population genetics [87,88,89]. Regions with more variations also can be utilized to develop candidate DNA barcodes and repeated sequences [79]. However, for some sect. *Bromatorrhiza* species, DNA barcode markers have been considered unidentifiable. For example, the frequently used chloroplast markers, including *rps16*, *trnL-trnF*, and *rpl32-trnL*, could not discriminate *A. hookeri*, *A. omeiense*, and *A. hookeri* var. *muliens* clearly [10]. Consequently, it is necessary to explore other areas of high variance that represent potential markers.

In this study, we performed comparative analyses of the 9 sect. *Bromatorrhiza* cp genomes and the 15 plastomes that included sect. *Bromatorrhiza*, and 10 different regions (e.g., *ndhF*, *ycf1*, *rpoB-trnC*) with high variation loci were obtained, respectively. The results showed that both groups contained *ndhF* and *ycf1* genes with high Pi values (Figure 9), and the other three intergenic regions (*psbI-trnS (UCC)-trnG (UCC)*, *atpI-rps2-rpoC2*, and *rpoB-trnC (GCA)*) with sub-high Pi values, indicating that these regions included more variable sites than other sequences. Based on this result and previous studies [37,69,89], *ndhF* and *ycf1* were identified as the optimal options of high-resolution molecular markers for species identification and phylogenetic studies of sect. *Bromatorrhiza* and its relatives, followed by these other three intergenic regions. In addition, it was observed that the Pi values of the 15 cp genomes were generally higher than those of the 9 sect. *Bromatorrhiza* plastomes. As more species were added to the analysis (from 9 to 15 species), the Pi values in the highly variable regions increased. A similar tendency was found in other studies [81,90]. Based on these results, we suggest that these five regions (*ndhF*, *ycf1*, *psbI-trnS (UCC)-trnG (UCC)*, *atpI-rps2-rpoC2*, and *rpoB-trnC (GCA)*) could be used as potential marker resources for species identification and phylogenetic studies in sect. *Bromatorrhiza*. Meanwhile, we still need to expand our research taxa to explore and validate these potentially highly divergent regions of DNA barcodes in depth to find more high-resolution DNA barcodes with a wide range of applicability.

### 4.3. Phylogenetic Analysis

The results of the phylogenetic analysis strongly support that *Allium* is monophyly, and three evolutionary lineages were detected (Figure 10), which is consistent with previous studies [7,10,37]. Based on phylogenetic analyses, we found that *A. kingdonii* was deeply nested within subgenus *Amerallium* Traub with strong support (PP = 1, BS = 100%), rather than within subgenus *Cyathophora* as previous studies suggested [7,10]. Huang et al. [11] also suggested that *A. kingdonii* is a member of subgenus *Amerallium*, which further supported our results. In addition, species with controversial systematic positions in previous studies were also resolved in our results. *A. cyathophorum*, *A*. *farreri*, *A. mairei*, and *A. spicatum* formed a monophyletic clade located in the subgenus *Cyathophora* rather than in the first evolutionary lineage as suggested by Xu [14], who classified *Allium* species based on their morphological characteristics. Li et al. [10] and Huang et al. [11] also proposed that *A. cyathophorum* and *A. farreri* should be moved out from sect. *Bromatorrhiza* and classified into the subgenus *Cyathophora* through chloroplast fragments (e.g., *rps16*, *rpl32-trnL*, and *trnL-F*). Thus, the systematic positions of the controversial species *A. kingdonii*, *A. cyathophorum*, and *A. farreri* are further solved here. Additionally, the position of *A. trifurcatum* was further confirmed. During the field survey and observation of specimens, we found that *A. trifurcatum* shares several similar biological characteristics with the species of sect. *Bromatorrhiza*, such as relatively thick and straight roots [14], but their molecular characteristics were remarkably different in this study. *A. trifurcatum* is located in the third evolutionary lineage and was a sister to *Allium ramosum* L. and *Allium tuberosum* Rottler ex Sprengle, which was strongly supported (PP = 1, BS = 100%). Our result is consistent with previous phylogenetic studies [10,11]. Moreover, the species relationships of sect. *Bromatorrhiza* were also uncovered. Previous molecular phylogenetic studies of *Allium* have involved only a relatively small number of species or populations in sect. *Bromatorrhiza* and mainly ITS sequences have been used, without convincing support for the monophyly of this section [7,10,23,91]. Here, our results strongly supported that sect. *Bromatorrhiza* is monophyletic, improved the support for phylogenetic trees through plastid genomes, and clear species relationships were detected. The *A. hookeri* complex was also confirmed and included five species [26], which shared high morphological resemblance [3]. However, the phylogenetic relationship between them is not well resolved and the systematic position of these species still needs further examination, especially between *A. chienchuanense*, *A. xiangchengense*, and *A. hookeri* var. *muliens* (Figure 10).

### 4.4. Selective Pressure Analysis

Species grow in different environments that are always subject to various climate factors, such as latitude, light, moisture, altitude, and temperature [92,93,94,95]. Genes associated with a particular environment are often assumed to be under positive selection [68], and this hypothesis has been broadly applied to detect genes associated with environmental adaptation [96,97]. Our results showed that the average Ka/Ks ratio was less than 1 for most genes. Former studies have shown that purifying selection can result in low rates of synonymous and non-synonymous DNA substitutions, such as in Aroideae [89] and *Paris* [98]. In addition, purifying selection was one of the most prevalent mechanisms of natural selection, which constantly eliminated harmful mutations [38]. For example, the gene *accD* encodes acetyl-CoA carboxylase, which has a role in fatty acid biosynthesis [99]; *rpl23* is used to synthesize the large ribosomal subunit [100]; *matK* encodes a maturase that is involved in splicing type II introns from RNA transcripts [101,102]; and the protein synthesized by *ndhA* occurs as a response to photo-oxidative stress [103]. All of these genes are important for plants’ adaption to the environment and survival. In our field investigation, sect. *Bromatorrhiza* species were mainly distributed in the QTP and HDM, growing on forest margins, mountain slopes, or grasslands at altitudes of 2100~3900 m. Therefore, the purifying selection of most chloroplast genes in sect. *Bromatorrhiza* species may be an evolutionary consequence of the maintenance of their adaptive traits.

Moreover, our results also found that the Ka/Ks values of three genes are more than 1 (*rps2*, *ycf1,* and *ycf2*) (Figure 11), and four genes (*atpF*, *atpI*, *rpl14*, and *rpoC2*) have positive selected sites (Table S12), which mean that these genes were subjected to positive selection. A previous study indicated that the product of the *rps2* gene plays an important role in defense signal transduction [104]. The *ycf1* gene encodes Tic214, a part of the translocator on the endosomal membrane (TIC) complex of *Arabidopsis thaliana* (L.) Heynh. chloroplasts, which affects plant survival [105], and the *ycf2* gene is also associated with adaptation in other species [106]. Other genes, including *atpF*, *atpI*, *rpl14*, and *rpoC2*, have also been detected under positive selection in other *Allium* species [37,39,40,44,97,107]. Therefore, these genes may have played key roles in the adaptation of species in sect. *Bromatorrhiza* during the evolution process.

## 5. Conclusions

In this study, we determined the complete chloroplast genome sequences of nine sect. *Bromatorrhiza* species using a de novo assembly approach. It is the first comprehensive systematic analysis to compare the plastome features and infer phylogenetic relationships using plastome data for sect. *Bromatorrhiza* and its relatives. Comparative analyses found that the plastomes of sect. *Bromatorrhiza* are conserved in terms of the genome structure, gene content and arrangement, SSRs, codon bias, and RNA editing sites but vary in their genome size and border of SC/IR. The plastid phylogenomic analyses demonstrate that plastome data are efficient and robust in improving the supports and resolutions of sect. *Bromatorrhiza* phylogeny and strongly support sect. *Bromatorrhiza* as a monophyletic group. In addition, five mutation hotspot regions were identified across the plastomes, which can serve as potential DNA barcodes for species identification between and within sect. *Bromatorrhiza*. Overall, our study enriches the data on the plastomes of sect. *Bromatorrhiza* and serves as a reference for subsequent phylogenomic studies of *Allium*.

## Figures and Tables

**Table 1:** Comparison of plastome features of the nine sect. *Bromatorrhiza* taxa.

Species	rbcL Accession	NCBI Accession	Length(bp)				GC Contents (%)				Number of Genes			
			Genome	LSC	SSC	IR	Genome	LSC	SSC	IR	Total	PCG	rRNA	tRNA
*A. wallichii*	KP207707	ON184003	152615	82283	17138	26597	37.0	34.9	30.3	42.5	134	88	8	38
*A. wallichii* var. platyphyllum	JX017625	ON184004	152926	82557	17121	26624	37.0	34.8	30.2	42.5	134	88	8	38
*A. macranthum*	JX017626	ON184005	152596	82168	17606	26411	37.1	34.9	30.4	42.7	134	88	8	38
*A. fasciculatum*	JX017623	ON184006	153214	82240	17406	26784	37.0	34.9	30.1	42.6	134	88	8	38
*A. xiangchengense*	JX017621	ON184007	152294	82378	17404	26256	37.0	34.8	30.1	42.7	134	88	8	38
*A. hookeri* var. muliens	JX017621	ON184008	153113	82366	17361	26693	37.0	34.8	30.1	42.6	134	88	8	38
*A. chienchuanense*	JX017621	ON184009	153549	82542	17357	26825	37.0	34.8	30.1	42.5	134	88	8	38
*A. omeiense*	JX017621	ON184010	153592	82609	17387	26798	37.0	34.8	30.0	42.6	134	88	8	38
*A. hookeri*	JX017621	ON184011	153682	82700	17386	26798	37.0	34.8	30.0	42.6	134	88	8	38

Note: LSC indicates large single-copy region; IR indicates inverted repeat region; SSC indicates small single-copy region; PCG indicates protein-coding genes.

## Data Availability

The complete chloroplast genome sequences of the nine species were deposited at NCBI (GenBank accession number: ON184003-ON184011).

## Supplementary Materials

The following supporting information can be downloaded at: https://www.mdpi.com/article/10.3390/genes13071279/s1, Figure S1: Analyses of RNA editing sites in 15 plastomes: (**A**) numbers of RNA editing sites distributed in different codon positions; (**B**) numbers of RNA editing sites presented in genes; Figure S2: Phylogeny of the 45 taxa inferred from maximum likelihood (ML) and Bayesian inference (BI) analyses based on shared CDSs; Figure S3: Pairwise Ka/Ks ratios in sect. *Bromatorrhiza*; Table S1: Sample collection information; Table S2: List of species and their accession numbers in GenBank included in this study; Table S3: List of genes present in the nine sect. *Bromatorrhiza* plastomes; Table S4: Numbers of SSR motifs identified in the 15 plastomes; Table S5: Number of different repeats of 15 plastomes; Table S6: Number of codons and RSCU values in 15 plastomes; Table S7: The statistics of the codon usage bias of 15 plastomes; Table S8: RNA editing site analyses of the 15 plastomes; Table S9: The genetic distance of 15 plastomes from each other; Table S10: The indel and SNP in the 15 plastomes; Table S11: Ka, Ks, and Ka/Ks values of genes in sect. *Bromatorrhiza* plastomes; Table S12: Positive selection analysis of the first lineage (clade 1) of Allium.
