# Supplementary material for: Comparative Analysis of the Complete Chloroplast Genomes in Allium Section Bromatorrhiza Species (Amaryllidaceae): Phylogenetic Relationship and Adaptive Evolution
Source: Genes (Basel). 2022 Jul 19;13(7):1279. doi: 10.3390/genes13071279 (PMC9324613; doi:10.3390/genes13071279)
Supplement: Supplementary file 1 [file genes-13-01279-s001.zip › Supplementary Figure S1-3.pdf]

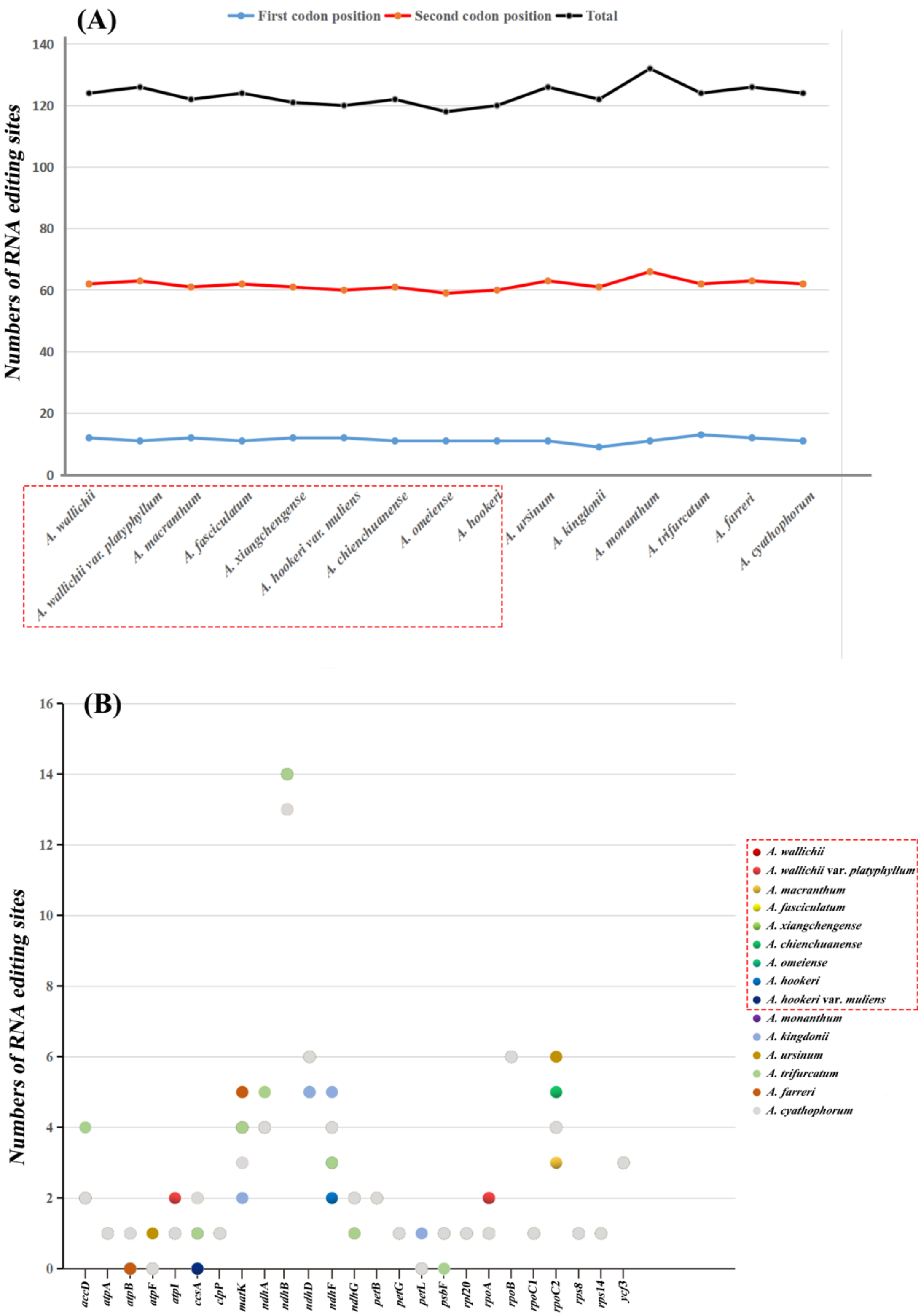

Additional file 1: Figure S1 Analyses of RNA editing sites in 15 plastomes: (A) numbers of RNA editing sites distributed in different codon positions; (B) numbers of RNA editing sites presented in genes.

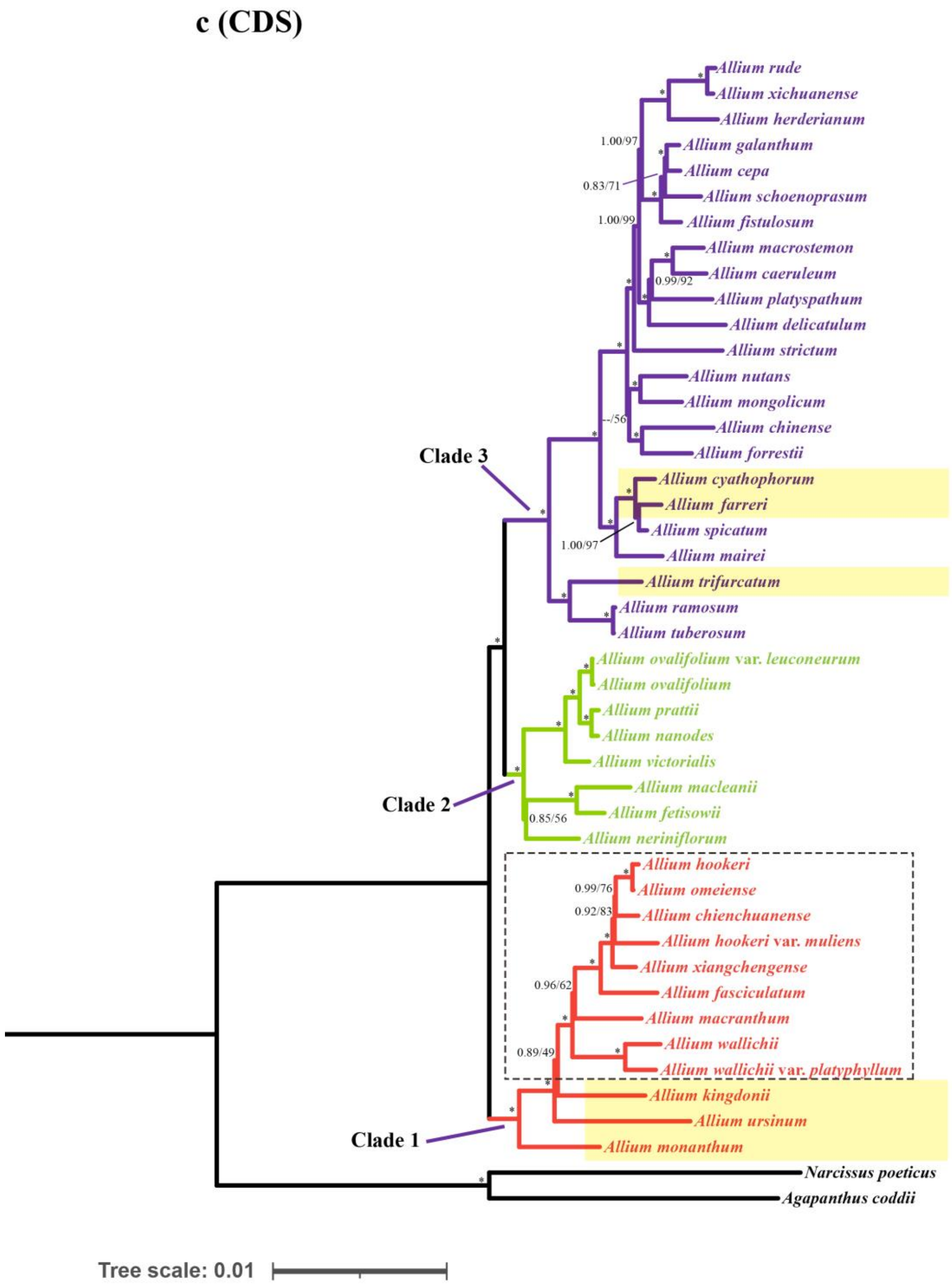

Additional file 2: Figure S2 Phylogeny of the 45 taxa inferred from Maximum likelihood (ML) and Bayesian inference (BI) analyses based on shared CDSs.

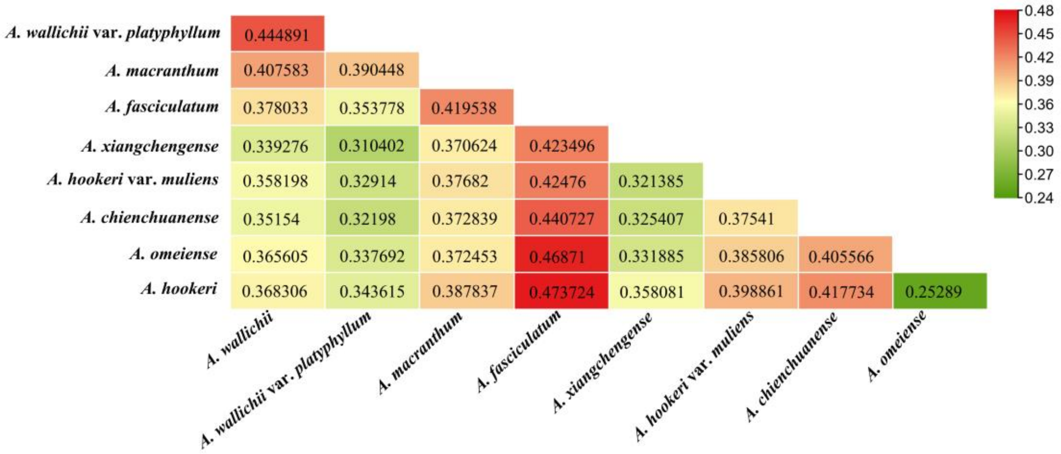

Additional file 3: Figure S3 Pairwise Ka/Ks ratios in sect. *Bromatorrhiza*. The heatmap shows pairwise Ka/Ks ratios between every sequence in the multigene nucleotide alignment.
